# History and Evolution of Yttrium-90 Radioembolization for Hepatocellular Carcinoma

**DOI:** 10.3390/jcm8010055

**Published:** 2019-01-07

**Authors:** Aman Saini, Alex Wallace, Sadeer Alzubaidi, M. Grace Knuttinen, Sailendra Naidu, Rahul Sheth, Hassan Albadawi, Rahmi Oklu

**Affiliations:** 1 Division of Vascular and Interventional Radiology, Laboratory for Minimally Invasive Therapeutics, Mayo Clinic, Phoenix, AZ 85054, USA; Saini.Aman@mayo.edu (A.S.); wallace.alex@mayo.edu (A.W.); alzubaidi.sadeer@mayo.edu (S.A.); knuttinen.grace@mayo.edu (M.G.K.); Naidu.Sailen@mayo.edu (S.N.); albadawi.hassan@mayo.edu (H.A.); 2 Department of Interventional Radiology, MD Anderson Cancer Center, Houston, TX 77054, USA; rasheth@mdanderson.org

**Keywords:** Yttrium-90, Y90, radioembolization, hepatocellular carcinoma, HCC, liver cancer

## Abstract

Hepatocellular carcinoma (HCC) is the most common form of primary liver cancer and affects millions worldwide. Due to the lack of effective systemic therapies for HCC, researchers have been investigating the use of locoregional tumor control with Yttrium-90 (Y90) radioembolization since the 1960s. Following the development of glass and resin Y90 microspheres in the early 1990s, Y90 radioembolization has been shown to be a safe and efficacious treatment for patients with HCC across Barcelona Clinic Liver Cancer (BCLC) stages. By demonstrating durable local control, good long term outcomes, and equivalent if not superior tumor responses and tolerability when compared to alternative therapies including transarterial chemoembolization (TACE) and sorafenib, Y90 radioembolization is being increasingly used in HCC treatment. More recently, investigations into variations in Y90 radioembolization technique including radiation segmentectomy and radiation lobectomy have further expanded its clinical utility. Here, we discuss the history and evolution of Y90 use in HCC. We outline key clinical trials that have established the safety and efficacy of Y90 radioembolization, and also summarize trials comparing its efficacy to existing HCC treatments. We conclude by reviewing the techniques of radiation segmentectomy and lobectomy, and by discussing dosimetry.

## 1. Introduction

**Figure 1:**
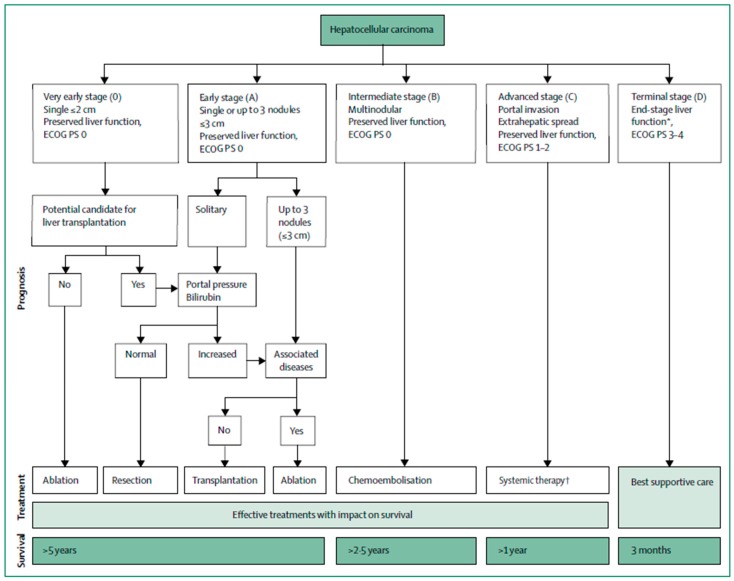
Barcelona Clinic Liver Cancer (BCLC) staging system and treatment algorithm The BCLC system provides treatment strategy in addition to prognostic outcomes by stage. Outcomes are expressed as median survival. ECOG PS = Eastern Cooperative Oncology Group Performance Status. * Patients with end-stage cirrhosis (i.e., Child-Pugh stage C, poor prognosis, or high Model for End-Stage Liver Disease (MELD) score). ^†^ First-line treatment with sorafenib or lenvatinib. Reproduced with permission from [6].

Hepatocellular carcinoma (HCC) is the most common form of primary liver cancer. Every year, there are approximately 700,000 new cases and 600,000 deaths attributable to HCC worldwide [1,2,3]. According to the Barcelona Clinic Liver Cancer guidelines, patients with very early or early stage disease are eligible for curative therapies including surgical resection or liver transplant (Figure 1). However, very few patients are eligible for these curative options due to advanced disease upon presentation. For unresectable early stage (BCLC A) patients, radiofrequency ablation (RFA) is recommended. In cases where ablation may be contraindicated, stage migration allows for treatment with the next best therapy, transarterial chemoembolization (TACE). For intermediate stage (BCLC B) patients, TACE is considered the standard of care. In recent years, radioembolization with Yttrium-90 (Y90) has emerged as an alternative to TACE. Further studies into the safety and efficacy of Y90 have led to its adoption across BCLC stages, with treatment goals ranging from complete tumor control to symptom palliation. Clinical practice guidelines, however, vary with regards to their position on radioembolization use. Certain working groups have advocated for radioembolization use in patients with unresectable liver-only or liver-dominant tumors with no clinical signs of liver failure, life expectancy of greater than 3 months, and with good performance status [4]. Conversely, the more recent European Association for the Study of the Liver (EASL) guidelines strongly recommend TACE for BCLC B patients, whereas Y90 radioembolization in BCLC B and C patients is not recommended due to failure to demonstrate a survival benefit when compared to TACE or sorafenib [5]. Despite several recent trials that have failed to show survival benefits of Y90 radioembolization versus TACE or sorafenib, the technique has demonstrated durable local control and a good safety profile, and ongoing trials are seeking to elucidate its optimal patient population. Here, we examine the history and evolution of Y90 radioembolization and summarize key studies that examine its safety, early efficacy, and long-term outcomes. We also review important clinical trials comparing Y90 to existing HCC therapies including RFA, TACE, and sorafenib. We conclude by addressing the concepts of radiation segmentectomy, lobectomy and Y90 radioembolization dosimetry considerations.

## 2. Early History of Y90 Radioembolization

The origins of hepatic artery-directed therapies for HCC can be traced back to the 1950s when investigators identified that hepatic tumors derive 80% of their blood supply from the hepatic artery, while normal hepatic parenchyma mostly receives its blood from the portal vein [7,8]. The following decade brought the first reports of Y90 radioembolization in hepatic malignancies. In 1965, Ariel described a technique for administration of Y90 from the celiac artery which was subsequently undertaken in four patients [9]. Complications were minimal, and the therapy offered symptomatic improvement for some patients. In 1967, Simon et al. reported on five patients with hepatic neuroendocrine tumors treated with Y90. Gastric toxicity was noted in two patients due to radiation-induced injury to the stomach, and overall outcomes were poor [10]. In 1973, the efficacy of Y90 resin microspheres for patients with colon cancer with metastatic disease to the liver was investigated. In a series of 25 patients, 17 were noted to have an objective decrease in tumor size [11]. These promising results spurred further studies over the following decade that would elucidate the optimal microsphere size needed for maximal tumor deposition. In a study on rats, Meade and colleagues determined that microspheres with diameters of 15 and 32.5 µm were 3 times more likely to lodge into hepatic tumors versus normal liver parenchyma [12]. Conversely, 50 µm microspheres were noted to be evenly distributed between normal and tumor tissue. Optimal microsphere size and tumor-to-liver distribution ratios were characterized subsequently [13]. The late 1980’s and early 1990’s brought a number of phase 1 trials [14,15] for the newly developed glass (TheraSphere, BTG International Group, London, UK) and resin microspheres (SIR-Spheres, Sirtex Medical Ltd., New South Wales, Australia). Several dose escalation studies demonstrated the safety and early efficacy of glass microspheres in patients with HCC [16,17,18], confirming the ability of this technique to selectively deliver high doses of radiation to tumors with acceptable toxicity. The results of these studies led to a humanitarian device exemption from the FDA in 1999 for the use of glass microspheres in patients with unresectable HCC. Furthermore, the results of safety and early efficacy studies for colorectal cancer patients treated with resin microspheres were also released [19,20]. A phase 3 clinical trial examining the efficacy of chemotherapy versus chemotherapy plus resin microspheres in the treatment of liver metastases from colorectal cancer demonstrated increased tumor response and progression free survival (PFS) in the combination group, leading to FDA premarket approval for resin microspheres for colorectal cancer liver metastases in 2002.

## 3. Seminal Y90 Radioembolization Studies

### 3.1. Safety, Efficacy, and Long-Term Outcomes

Y90 radioembolization’s role in HCC was first explored in the setting of portal vein thrombosis (PVT). It is estimated that approximately 1 in 3 HCC patients will develop PVT [21] and historically, TACE in patients with PVT was relatively contraindicated out of concern for iatrogenically induced acute liver failure unless superselective or segmental embolization could be achieved. In 2004, Salem and colleagues reported on the safety of Y90 glass microspheres in unresectable HCC patients with PVT [22]. In a cohort of 15 patients with PVT of one or both first order segmental portal vein branches who underwent Y90 radioembolization, radioembolization was well-tolerated with grade 1-2 bilirubin toxicity noted in only 5 patients. Importantly, this increase in post-treatment bilirubin was attributed to intrahepatic disease progression rather than treatment effect. No patients experienced liver failure, and for the first time, Y90 radioembolization was confirmed to be safe in HCC patients with PVT. Furthermore, it was found that due to the minimally embolic nature of Y90 glass microspheres, treatment with this form of radioembolization did not preclude future treatments with other arterial therapies such as TACE or hepatic artery infusion chemotherapy, paving the way for investigations into optimal treatment sequencing [22]. In a similar two-center phase 2 study in 2008, the safety and efficacy of Y90 radioembolization using glass microspheres in 108 unresectable HCC patients with and without PVT of the branch or main portal vein was investigated [23], building upon previous results in this patient population. For all patients, there were no cases of radiation-induced gastritis or pneumonitis. Post-treatment elevations in bilirubin and development of ascites were higher in the group with main PVT. Importantly, there was no increased risk of hepatic failure or encephalopathy between patients with main PVT versus those with branch or no PVT. Moreover, there were no significant treatment-related complications or treatment-related deaths. With respect to efficacy, partial responses were noted in 42.2% of patients using WHO criteria and in 70% of patients using EASL criteria. In 2013, treatment sequencing of HCC patients with PVT was further elucidated. In a cohort of 63 patients treated with Y90 radioembolization who experienced disease progression, investigators noted that 64% of patients were deemed ineligible for systemic treatment or clinical trials due to worsened Child-Pugh status (Child Pugh B or C) at time of progression [24]. Additionally, survival was significantly shorter when comparing Child-Pugh A versus B groups (13.8 vs. 6.5 months). These findings led investigators to conclude that an adjuvant approach to sequencing, where systemic treatment is administered after Y90 radioembolization but before disease progression/declines in liver function, may be beneficial in this patient population [24].

With the safety profile of Y90 radioembolization better understood and its efficacy coming to light, several studies began to examine long-term treatment outcomes across stages. In the first long term outcomes analysis for 291 HCC patients treated with Y90 radioembolization using glass microspheres, Salem et al. measured response rate, time to progression (TTP), and survival across stages [25]. The investigators noted objective response rates (ORRs) of 42% and 57% based on WHO and EASL criteria, respectively. TTP for the entire patient cohort was 7.9 months and varied by Child-Pugh stage, as did survival outcomes. Patients with Child-Pugh A disease survived significantly longer than those with Child-Pugh B disease (17.2 vs. 7.7 months). Additionally, patients with Child-Pugh B disease and PVT had the worst outcomes with a median OS of 5.6 months. This study was the first to demonstrate the varying TTP and outcomes by Child-Pugh status. In a similar study of 108 intermediate-advanced HCC patients undergoing radioembolization with glass microspheres in Europe, TTP for the entire cohort was 10 months, and the median OS was 16.4 months [26]. These outcomes compared favorably to the outcomes of similar patients treated with sorafenib monotherapy in the SHARP trial (TTP 5.5 months, median OS 10.7 months) [27], setting the stage for combination studies discussed subsequently. In 2013, Mazzaferro and colleagues conducted the first phase 2 study examining the efficacy and long term outcomes of Y90 radioembolization in BCLC intermediate and advanced HCC [28]. In 52 patients with a median follow up time of 36 months, the median TTP was 11 months and was not significantly different for patients with PVT versus those without PVT. Additionally, the median OS was 15 months and objective responses were noted in 40.4% of patients. These results were concordant with previous studies of radioembolization in intermediate-advanced HCC patients with or without PVT, leading researchers to conclude that intraarterial Y90 is a safe, well-tolerated, and efficacious treatment for these patients. More recently, a retrospective analysis by Gordon et al. sought to elucidate baseline patient characteristics and prognostic factors in unresectable HCC patients termed “Super Survivors”—those remaining alive >3 years after Y90 treatment [29]. Sixty-seven “Super Survivors” were identified; surprisingly, they spanned BCLC stages A-D and 40% had multifocal disease. Upon further analysis, the common variable among these patients was an imaging response after treatment. Furthermore, patients undergoing segmental Y90 radioembolization were noted to have increased OS when compared to those undergoing lobar treatment. One possible confounder that may have influenced these results was crossover to alternate locoregional therapy after progression of disease. Nevertheless, imaging response to Y90 radioembolization treatment may be a favorable prognostic factor. In summary, over the past decade, Y90 radioembolization has demonstrated itself to be a safe and effective treatment for patients with unresectable HCC across clinical stages.

### 3.2. Comparisons to Existing Therapies

In recent years, there have been several studies comparing the efficacy of Y90 radioembolization to TACE and other locoregional therapies in the settings of definitive treatment and in downstaging as a bridge to transplant. In 2016, a systematic review and meta-analysis of 5 studies and 553 patients with unresectable HCC who underwent TACE or Y90 radioembolization was conducted [30]. The analysis showed no significant survival differences for up to 4 years between the groups. Additionally, partial and complete response rates were similar, as were the complication profiles between the two treatments, although patients receiving TACE were noted to have increased post-treatment pain [30]. These results differed from other meta-analyses around the same time, likely due to the retrospective nature of these analyses [31,32]. In 2016, the results of the PREMIERE trial, a landmark phase 2 study comparing the TTP for 45 BCLC A and B patients randomized to TACE or Y90 were released [33]. The investigators discovered a significantly longer median TTP for patients receiving Y90 radioembolization when compared to TACE (26 months vs. 6.8 months) although no differences in survival were noted, “suggesting local control is insufficient for survival improvement in cirrhotic patients with competing risks of death” [33]. It should be noted that the trial was closed early due to slow accrual. Despite the lack of survival benefits, the researchers did note that the increased TTP and improved local control for patients treated with Y90 could decrease transplant list drop-out. Furthermore, lower rates of diarrhea and hypoalbuminemia, improved quality of life [34], and outpatient delivery potentially make Y90 an attractive alternative to transplant-eligible HCC patients when compared to TACE. In the setting of downstaging for transplant eligibility, a comparative analysis of TACE versus Y90 demonstrated the superior performance of Y90 in downstaging HCC patients from United Network for Organ Sharing (UNOS) T3 to T2 [35]. In this study of 86 patients, partial response rates were significantly higher in the Y90 patients, and downstaging was achieved in more Y90 patients when compared to TACE (58% versus 31%). By more efficiently downstaging patients from T3 to T2 and thus placing them within the confines of the Milan criteria, Y90 radioembolization may more quickly allow for UNOS priority status upgrade and quicker access to donor organs.

In addition to comparisons with TACE, the efficacy of Y90 radioembolization as a bridge to transplant has also been compared to stereotactic body radiation therapy (SBRT) and RFA. In a retrospective study of 60 HCC patients undergoing Y90, TACE, RFA, and SBRT, Mohammed et al. determined Y90 radioembolization to have the highest rates of pathologic complete response among the locoregional therapies (Y90/TACE/RFA/SBRT: 75%/41%/60%/28.5%) [36]. Radiologic responses were also highest in the Y90 group at 33%, although this was not statistically significant. Finally, Grade 3 and 4 toxicities were significantly higher for patients receiving TACE and RFA. Although the choice of local therapy depends on numerous tumor and patient specific factors, Y90 has demonstrated itself to be an effective and tolerable local therapy for downstaging patients as a bridge to transplant [37].

Several clinical trials have evaluated the role of Y90 radioembolization in combination with sorafenib. In 2014, Kulik and colleagues performed a phase 1 randomized trial to assess the safety and adverse event profile of Y90 radioembolization plus sorafenib compared to Y90 alone in 20 HCC patients awaiting a liver transplant [38]. In the combination therapy group, all patients required dose reductions in sorafenib due to gastrointestinal and dermatologic side effects while 3 patients ultimately discontinued the drug. Importantly, in the combination group, peri-transplant (<30 day) biliary complications in four patients and acute cellular rejection (3/8 patients) were observed. These unexpected findings led the investigators to conclude that caution should be exercised when administering sorafenib plus Y90 radioembolization in the transplant setting. Additionally, survival rates were not significantly different between the two groups at 3 years (70% in monotherapy group, 72% in combination therapy group).

The relative efficacy of Y90 radioembolization versus sorafenib for advanced HCC patients was evaluated in the SARAH trial [39]. This was a phase 3, randomized, controlled, open-label, multicenter trial that included 459 locally advanced HCC patients (BCLC C) or those previously treated with two unsuccessful rounds of TACE. Patients were randomized to sorafenib or Y90 radioembolization using resin microspheres and the primary end point was OS, with secondary endpoints including PFS, TTP, response rate, adverse events and quality of life (QOL). The median OS was not significantly different between the two treatment arms, with patients surviving 8.0 months in the radioembolization group and 9.9 months in the sorafenib group. Median PFS was similar between the two groups in both the intention-to-treat (ITT) population and in the per-protocol population. The objective response rate (ORR) was significantly higher in the Y90 radioembolization intention to treat population. Furthermore, higher rates of treatment related adverse events including fatigue, hematologic abnormalities, diarrhea, abdominal pain, and dermatologic reactions were noted in the sorafenib group.

More recently, a randomized, phase 3, open-label, multicenter trial comparing sorafenib to Y90 radioembolization using resin microspheres in 360 patients with locally advanced HCC patients with and without vascular invasion was conducted (SIRveNIB) [40]. The primary endpoint was OS and secondary endpoint was PFS, tumor response rate, toxicity and QOL. Median OS was not statistically different between the two groups (8.8 versus 10.0 months in the Y90 radioembolization and sorafenib groups, respectively), although patients treated with Y90 radioembolization were noted to have higher tumor response rates and two-fold fewer adverse events. These results are similar to the SARAH study—both studies were negative and demonstrate no survival benefits for Y90 radioembolization when compared to sorafenib. Finally, it is worth noting that although these phase 3 trials measured injected radiation dose, actual dose delivered to the tumor was not measured, which has been shown to predict treatment response [41]. Increased focus on Y90 radioembolization dosimetry moving forward may help to better elucidate treatment outcomes.

Preliminary results of a randomized, controlled, phase 2 trial comparing the safety and efficacy of sorafenib plus Y90 radioembolization using resin microspheres versus sorafenib alone in 529 patients with unresectable locally advanced HCC not amenable to TACE have been released (SORAMIC). The primary endpoint was OS and although median OS rates did not significantly differ between the two treatment groups in both the ITT and per-protocol population, possible survival benefits were noted in subgroups of younger patients (<65 years old), those with non-alcoholic cirrhosis, and those without cirrhosis at all [42]. These initial results support further investigation into the optimal patient population to be treated with this combination therapy.

## 4. Variations in Y90 Radioembolization Technique

### 4.1. Radiation Segmentectomy

**Figure 2:**
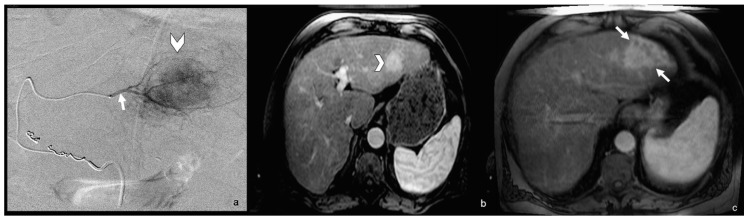
Radiation segmentectomy of HCC. (**a**) Angiogram of segmental tumor-supplying vessel (arrow) and tumor (arrowhead). (**b**) Pre-treatment MRI demonstrating arterial-enhancing tumor (arrowhead). (**c**) Post-treatment MRI showing radiation-induced changes in tumor-containing segment (arrows). Reproduced with permission from [43].

Tumor resection or ablation are considered curative therapies for patients with HCC ≤3 cm. At times, however, ablation is not feasible given tumor size or the proximity to adjacent structures such as the biliary tree or vasculature. In recent years, research into the concept of radiation segmentectomy, or superselective radioembolization via infusion of a calculated lobar dose into a segmental vessel, has shed light on the suitability of this treatment for patients with small lesions confined to ≤2 liver segments (Figure 2). This technique allows for dose escalation to the tumor, while minimizing radiation exposure to adjacent healthy parenchyma. In the initial study describing this technique, researchers were able to selectively deliver Y90 microspheres to ≤2 segments of the liver, with median doses of 512 Gy and 210 Gy assuming uniform and non-uniform distributions, respectively [43]. These ultra-high doses of radiation greatly exceeded the tumoricidal dose of 120 Gy to the injected liver volume for HCC treated with glass microsphere radioembolization. In this particular study, tumor response was excellent with response rates of 59% and 81% using WHO and EASL guidelines, respectively. Importantly, toxicities remained low, and no patients exhibited signs of radiation induced liver disease (RILD). The results of this study led to a larger, multicenter analysis of patients with treatment-naïve solitary HCC ≤5 cm treated with radiation segmentectomy [44]. For 102 patients, complete response, partial response, and stable disease were noted in 47%, 39%, and 12% of patients, respectively, using modified Response Evaluation Criteria in Solid Tumors (mRECIST). Median TTP was 33.1 months. Furthermore, close to 1/3 of patients underwent transplant following radiation segmentectomy, allowing for radiology-pathology correlation. Pathology revealed 90–100% pathological necrosis for all patients with improved necrosis noted when the dose exceeded 190 Gy. The results of these studies were confirmed in a recent analysis of long term outcomes for solitary HCC patients with lesions ≤5 cm treated with radiation segmentectomy [45]. In this study which included patients treated over 14 years, median TTP was 2.4 years and median OS was 6.7 years. Furthermore, the 5-year overall survival probability was 75%, which is comparable to 5-year survival for patients undergoing other curative treatment options including resection, RFA ablation, or transplant.

Investigators recently compared outcomes for radiation segmentectomy to segmental TACE. In a retrospective analysis including 108 patients with localized HCC not amenable to resection or ablation, radiation segmentectomy and segmental TACE were compared [46]. Overall response rates were higher for radiation segmentectomy versus TACE (84% vs. 58%). Median PFS was also higher in the radiation segmentectomy group (564 days vs. 271 days), as were rates of local control (92% vs. 70%). However, the OS rates were not different between the two groups. These results parallel those from a more recent study in which radiation segmentectomy was compared to segmental TACE for solitary HCC <3 cm [47]. Although there was no trend towards increased survival for Y90 patients, response rates were comparable and radiation segmentectomy did result in longer time to second treatment.

### 4.2. Radiation Lobectomy

**Figure 3:**
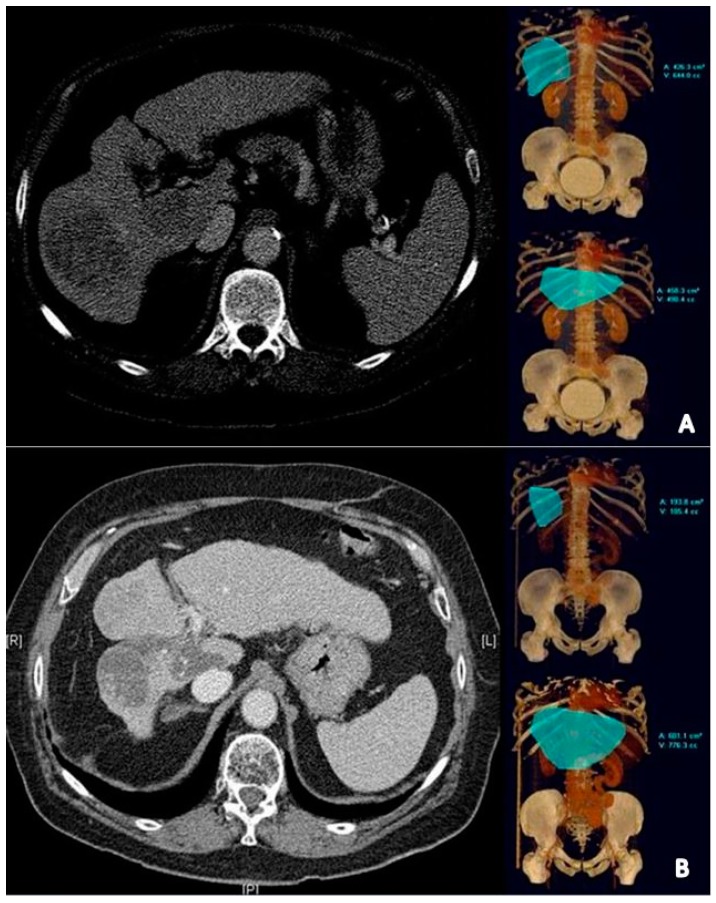
Pre- and post-treatment radiation lobectomy imaging. (**A**) Pre-treatment arterial phase contrast-enhanced CT showing multifocal right lobe HCC. Pre-treatment right and left HLVs are 644 and 498 mL, respectively. (**B**) Post-treatment contrast-enhanced CT demonstrates right lobe atrophy, tumor necrosis, and left lobe hypertrophy. Post-treatment HLVs are 185 mL and 776 mL, respectively. Reproduced with permission from [49].

Radiation lobectomy is a variation on the conventional radioembolization technique that seeks to take advantage of the volumetric liver changes associated with lobar radioembolization, specifically the ipsilateral lobar atrophy and contralateral lobar hypertrophy. It has been described in patients who may be candidates for curative surgical resection but are excluded from surgery because of a small future liver remnant (FLR). Similar volumetric changes have been observed after portal vein embolization (PVE), which has been the standard of care as a bridge to resection in patients with ipsilateral lobe tumors [48]; however unlike PVE which simply redirects portal blood, radiation lobectomy additionally and simultaneously attempts to obtain tumor control, possibly limiting tumor progression into the healthy contralateral lobe. Radiation lobectomy was first described in 2009 in a cohort of 20 HCC patients, all of whom had unilobar right sided tumors [49]. For right lobe tumors treated with Y90 radioembolization, a 52% reduction in right hepatic lobar volume (HLV) was noted, while left HLV decreased by 40% (Figure 3). These volumetric decreases were inclusive of both normal parenchyma and tumor, indicating tumor control and true parenchymal atrophy. Many of these patients went on to receive a transplant or resection and 5-year OS was noted to be 46%, which is comparable to 5-year survival rates for patients receiving upfront curative resection [50]. Given that the radiation doses administered to these patients were not significantly different from traditional radioembolization doses (80–150 Gy), the authors hypothesized that the “lobar atrophy-hypertrophy complex” most probably results from the redirection of blood from a fibrotic, radiation-treated hepatic lobe—similar to PVE, except with the added benefits of concomitant tumor control [49]. Another important takeaway from this study is that of laterality and treatment selection—given the small contribution of the left hepatic lobe to total liver volume and thus the increased likelihood of upfront resection in patients with left lobe tumors, patients with right lobe tumors may be more likely to undergo Y90 and/or radiation lobectomy—a phenomenon seen in this study consisting of only right lobe tumors [49].

In a follow up analysis by the same study group, investigators performed a time dependent analysis on the future liver remnant volume in 67 unresectable HCC patients [51]. The median % FLR hypertrophy reached 45% at 9 months and the median maximal % FLR hypertrophy was 26%. Although the kinetics of hypertrophy in this study were slower than the kinetics of PVE reported in other studies, synchronous tumor control potentially offered a significant advantage. In this study, significant reductions in median tumor volume at 3 months, from 134 cc to 99 cc, were observed. At 9 months, median tumor volume was 56 cc, indicative of adequate tumor control while awaiting contralateral lobe hypertrophy. Furthermore, PVE is generally contraindicated in HCC patients with PVT. However, in this analysis, patients with PVT treated with radiation lobectomy actually exhibited greater hypertrophy, which is probably explained by further increases in diversion of portal blood flow to the contralateral lobe. Importantly, progression of disease into the left lobe occurred in 19% of patients, which is comparable to rates of local recurrence at 1-year for early stage patients treated with RFA [52]. In summary, given its ability to induce FLR hypertrophy with simultaneous tumor control, radiation lobectomy offers a potential alternative to PVE in the bridge to resection setting.

### 4.3. Dosimetric Considerations

As with external beam radiotherapy, the tumor response, toxicity and survival outcomes of radioembolization stem from the ability to deliver a tumoricidal dose of radiation to a hepatic tumor, while simultaneously minimizing radiaiton exposure to adjacent healthy parenchyma. Given the variability in patient responses to radioembolization and recent negative phase 3 studies which measured injected radiation dose and not the dose delivered to the tumor, much attention is being placed on radioembolization dosimetry. In 2013, Garin et. al. evaluated the impact of dosimetry based upon Technetium-99m Macroaggregated Albumin Single Positron Emission Computed Tomography (MAA SPECT/CT) in predicting treatment response, toxicity and survival for 71 patients with inoperable HCC treated with glass microsphere radioembolization [53]. MAA SPECT/CT was used to calculate absorbed tumor dose (TD), healthy injected liver dose (HILD), and total injected liver dose. The investigators noted median TD’s of 342 Gy and 191 Gy for responding and non-responding lesions, respectively. Furthermore, in patients with a TD < 205 Gy, the TTP and median OS were 5.5 months and 11.5 months, respectively, whereas for patients with TD > 205 Gy, the TTP and median OS were 13.0 and 23.2 months, respectively. Based on pre-treatment TD and HILD, select patients including those with large lesions or PVT were able to undergo treatment intensification without added toxicity, resulting in good clinical outcomes [53]. A similar analysis using MAA SPECT/CT was recently performed in a cohort of 184 patients from the SARAH trial [54]. Patients who received TD > 100 Gy had significantly longer survival than those receiving <100 Gy (14.1 vs. 6.1 months). Disease control was also signficantly associated with higher TD, highlighting the predictive value of this dosimetric method and need for improved tumor dosimetry in clinical trials moving forward. With regards to the technique of radiation lobectomy, dosmietric parameters on MAA SPECT/CT have been associated with signifcantly higher maximal hypertrophy of an untreated lobe. In an analysis of 73 HCC patients with unilobar disease undergoing radioembolization with glass microspheres, Palard et. al. determined that maximal hypertrophy of an untreated lobe ≥ 10% occurred signficantly more in patients treated with HILD ≥ 88 Gy, when compared to patients treated with <88 Gy [55]. These results, if validated in larger cohorts, could help to guide radiation lobectomy planning. More recently, three-dimensional voxel-based dosimetry for patients undergoing radioembolization with resin microspheres has also been described and shown to be an independent factor associated with tumor control and clinical outcomes [56]. Collectively, these advances have the potential to personalize dosimetry and improve clinical outcomes for patients treated with radioembolization.

## 5. Summary

Since the 1960s, investigators have been studying the safety and efficacy of Y90 radioembolization for HCC. With the introduction of glass and resin Y90 microspheres in the early 1990s, numerous trials were initiated to further explore the utility of radioembolization in hepatic malignancies. Y90 is a versatile tool with multiple uses including downstaging of patients for curative treatments, providing tumor control as a bridge to transplant or resection, curative therapy when used as segmentectomy, and left lobar hypertrophy in the setting of radiation lobectomy. Because of this versatility, Y90 radioembolization has a potential role for HCC patients across BCLC stages.
